# *Helicobacter pylori* Eradication Therapy Affect the Gut Microbiota and Ghrelin Levels

**DOI:** 10.3389/fmed.2021.712908

**Published:** 2021-08-12

**Authors:** Gracia Mª Martín-Núñez, Isabel Cornejo-Pareja, Mercedes Clemente-Postigo, Francisco J. Tinahones, Isabel Moreno-Indias

**Affiliations:** ^1^Department of Endocrinology and Nutrition, Instituto de Investigación Biomédica de Málaga (IBIMA), Hospital Universitario Virgen de la Victoria, Málaga, Spain; ^2^Centro de Investigación Biomédica en Red de Fisiopatología de la Obesidad y la Nutrición (CIBEROBN), Instituto de Salud Carlos III, Madrid, Spain; ^3^Department of Cell Biology, Physiology, and Immunology, Maimónides Biomedical Research Institute of Córdoba (IMIBIC)/University of Córdoba/Reina Sofia University Hospital, Córdoba, Spain

**Keywords:** *Helicobacter pylori*, gut microbiota, ghrelin, eradication treatment, antibiotic

## Abstract

**Background:** Antibiotic therapy used to eradicate *Helicobacter pylori* has been associated with changes in plasma ghrelin and alterations in the gut microbiota. On the other hand, changes in ghrelin levels have been related to changes in gut microbiota composition. Our aim was to evaluate the relationship between changes in the gut microbiota and ghrelin levels in *H. pylori* infected patients who received antibiotic treatment for its eradication.

**Methods:** A prospective case-control study that included forty *H. pylori*-positive patients who received eradication therapy (omeprazole, clarithromycin, and amoxicillin) and twenty healthy *H. pylori* antigen-negative participants. Patients were evaluated, including clinical, anthropometric and dietary variables, before and 2 months after treatment. Gut microbiota composition was analyzed through 16S rRNA amplicon sequencing (IlluminaMiSeq).

**Results:** Changes in gut microbiota profiles and decrease in ghrelin levels were identified after *H. pylori* eradication treatment. Gut bacteria such as *Bifidobacterium longum, Bacteroides, Prevotella, Parabacteroides distasonis*, and *RS045* have been linked to ghrelin levels fasting and/or post meals. Changes in the abundance of *Lachnospiraceae*, its genus *Blautia*, as well as *Prevotella stercorea*, and *Megasphaera* have been inversely associated with changes in ghrelin after eradication treatment.

**Conclusions:** Eradication treatment for *H. pylori* produces changes in the composition of the intestinal microbiota and ghrelin levels. The imbalance between lactate producers such as *Blautia*, and lactate consumers such as *Megasphaera, Lachnospiraceae*, or *Prevotella*, could trigger changes related to ghrelin levels under the alteration of the eradication therapy used for *H. pylori*. In addition, acetate producing bacteria such as *B. longum, Bacteroides*, and *P. distasonis* could also play an important role in ghrelin regulation.

## Introduction

Ghrelin, known as the hunger hormone, is predominantly produced in the stomach (1), but it is also produced in other organs such as the pituitary, hypothalamus, adrenal gland, placenta, and pancreas (2, 3) as well as the small and large intestines (4). *Via* binding to the growth hormone secretagogue receptor 1a (GHSR1a), ghrelin performs multiple physiological functions, including the stimulation of food intake, growth hormone secretion (GH) (5), adiposity (6), gastric motility, acid secretion (7), and insulin secretion inhibition (8). Serum ghrelin concentrations increase during fasting (when the stomach is empty), and decrease after eating (9). Moreover, ghrelin levels have been related to body mass index (BMI) in a complicated manner, even showing a certain level of ghrelin-resistance (6, 10).

The gastrointestinal microbiota have the potential to modulate the energy metabolism by altering hormone levels affecting their secretion capacities (11) or through the intestine-brain axis (12). The interaction between the microbiome and ghrelin was corroborated by findings showing that germ-free mice have different ghrelin concentrations than conventionalized mice (13). Experimental studies revealed changes in circulating ghrelin levels linked to changes in gut microbiota composition, suggesting that the ghrelinergic system may be under regulation of the gut commensal microbes (14).

*Helicobacter pylori* is known to infect the gastric mucosa, induce inflammation, and alter both gastric and intestinal microbiota resulting in a broad spectrum of alterations, including metabolic syndrome-related disorders (15). At the same time, the therapeutic strategies used to eradicate *H. pylori* have also been associated with alterations in the gut microbiota (16). However, there are currently only several studies relating alterations in the gut microbiota by *H. pylori* and its eradication with changes in metabolic parameters. Our group have previously published alterations in the microbiota due to *H. pylori* infection and eradication therapy, and more importantly, these alterations have been related to glucose metabolism (17), GLP-1 levels (18), and blood lipid levels (19).

*H. pylori* eradication with antimicrobials has been associated with changes in plasma ghrelin (20–22), and weight gain (23). Moreover, *H. pylori* may alter ghrelin levels through the induction of damage to endocrine hormone-producing cells from the gastric mucosa (24), but also through the alteration of the gut microbiota composition (25). However, the relationship between gut microbiota and ghrelin levels after *H. pylori* therapy has been little explored (26). Accordingly, our objective was to study the relationship between the gut microbiota and ghrelin levels in *H. pylori* infected patients who received antibiotic treatment for its eradication.

## Materials and Methods

### Study Subjects and Design

Forty patients with positive *H. pylori* antigen in stool determined by immunochromatography and 20 healthy adults (control group) were recruited. The inclusion criteria were: (1) age 18–65 years, and (2) first *H. pylori* infection (for positive *H. pylori*). Exclusion criteria were (1) type 1 or 2 diabetes diagnosis; (2) prior documented treatment of *H. pylori*; (3) antibiotic use within 3 months previous to enrollment; (4) informed consent could not be obtained; (5) eradication therapy failure. Sample size was assessed considering a reduction in richness of 16% because of the antibiotic therapy based on previous microbiota studies (27, 28) and a pilot study (non-published).

The study included two visits, before and 2 months after treatment (omeprazole 20 mg, clarithromycin 500 mg, amoxicilin 1,000 mg twice daily for 10 days) for patients and only one visit for the control group. All visits included a physical examination, a fasting blood sample, and a 75 g oral glucose tolerance test (OGTT) at 30, 60, and 120 min.

Also, stool samples were collected during each visit and frozen at −80°C until DNA extraction. The study protocol was approved by the Medical Ethics Committee at Virgen de la Victoria University Hospital and conducted in accordance with the Declaration of Helsinki. Written informed consent was provided by all participants, who also were verbally informed of the characteristics on the study.

### Anthropometric, Biochemical, and Dietetics Measurements

Body weight, height, and waist circumferences were measured according to standardized procedures (29). Total cholesterol (mg/dl), high-density lipoprotein (HDL) cholesterol (mg/dl), triglycerides (mmol/L), and serum glucose were measured using a standard enzymatic method (Randox Laboratories Ltd.). Low-density lipoprotein (LDL) cholesterol (mg/dl) was calculated by using the Friedewald formula. Plasma insulin (pmol/L) was assessed by electrochemiluminescence (E170 module, Roche Diagnostics). Plasma glucagon-like peptide-1 (GLP-1) (ng/mL) and total ghrelin levels (pg/mL) were measured manually using commercial kits (human GLP-1 EIA Kit, GENTAUR Belgium, Kampenhout, Belgium, and Human Ghrelin Fluorescent EIA Kit, Phoenix Pharmaceuticals, Burlingame, CA, USA, respectively) and expressed in ng/mL and pg/mL, respectively. The area under the ghrelin curve (ghrelin AUC) (pg^*^min/mL) was calculated from the serum ghrelin concentrations at time points 0, 30, 60, and 120 min after the oral glucose tolerance test, using the trapezoidal rule. Food intake was evaluated by using seven 24-h dietary recalls for case and control groups. Total energy (kcal/day), macronutrients [proteins, fats, total carbohydrates, dietary fiber, and sugars (g/day)] and micronutrients [total polyphenols (mg/day)] for each participant were obtained using DIAL nutrition program and the professional Diet Balancer software (Cardinal Health Systems Inc.).

### Gut Microbiota Analysis

The determination of the microbiota has been described in detail in a previous study (17). Briefly, the fecal bacterial microbiota composition was determined using tag-encoded 16S rRNA gene Miseq-based (Illumina, CA, USA) high throughput sequencing. The 16S rRNA V3-V4 amplicon (amplicon size ~460 bp) was amplified by polymerase chain reaction (PCR) using the universal primers reported by Klindworth et al. (30). Dual indices and Illumina sequencing adapters were attached to sequence the amplicons, using the Nextera XT Index Kit (Illumina, CA, USA). Paired-end sequencing of amplicons was conducted on the Illumina MiSeq platform using the v3 kit generating 2 × 301 nucleotide reads (Illumina, San Diego, USA).

The merged paired-end reads were analyzed using the Quantitative Insights Into Microbial Ecology (Qiime) tool (version 1.9.1, open source software). The operational taxonomic units (OTUs) were generated by clustering sequences with 97% similarity and the representative sequences, selected as the most abundant in each cluster, underwent taxonomic alignment by UCLUST consensus (http://drive5.com/usearch/manual/uclust_algo.html) to obtain the taxonomic assignment and relative abundance of each OTU using the Greengenes 16S rRNA gene database (http://greengenes.lbl.gov/cgi-bin/nph-index.cgi). Raw data can be found in the SRA database public repository from NCBI within the BioProject accession number PRJNA517270.

### Statistical Analysis

The statistical analysis was performed with SPSS 22.0 (SPSS Inc., Chicago, IL, USA) and QIIME (version 1.9.1; open source software). The data were expressed as mean ± standard deviation. Statistical comparisons between the means for independent samples and paired samples (pre- and post-eradication treatment) were performed using the Student's *t*-test. Non-parametric variables were evaluated by Mann–Whitney and Wilcoxon signed-rank tests. The correlation between quantitative variables including analytical, clinical, and microbial populations was analyzed using the Spearman bivariate correlations test. Linear regression models (both univariate as multivariate adjusted by age, sex, and BMI) were applied to identify bacterial changes as independent predictors of the selected variables (ghrelin levels and AUC grhelin). Statistical significance was established at *p* < 0.05. *P*-values were corrected for multiple comparisons using the Benjamini–Hochberg method when appropriate.

## Results

### General Characteristics

The anthropometric and clinical characteristics of the subjects included in this study have been previously described elsewhere (17). In summary, no significant differences were found in BMI, waist circumference, plasma glucose, plasma insulin, and triglycerides between study groups. However, HDL cholesterol and GLP-1 levels increased significantly (55.36 ± 16.36 vs. 52.97 ± 12.9, *p* = 0.021 and 4.2 ± 0.4 vs. 3.6 ± 0.3; *p* < 0.001, respectively) in patients after *H. pylori* eradication with antibiotic therapy, while LDL cholesterol levels increased in *H. pylori*-infected subjects compared to controls (121.45 ± 35.8 vs. 102.05 ± 34, *p* = 0.036) (17). There were no statistically significant differences in dietary intake of energy and macro or micronutrients, as well as in dietary fiber (*p* > 0.05) between the study groups (data not shown).

### Ghrelin Levels

**Figure 1:**
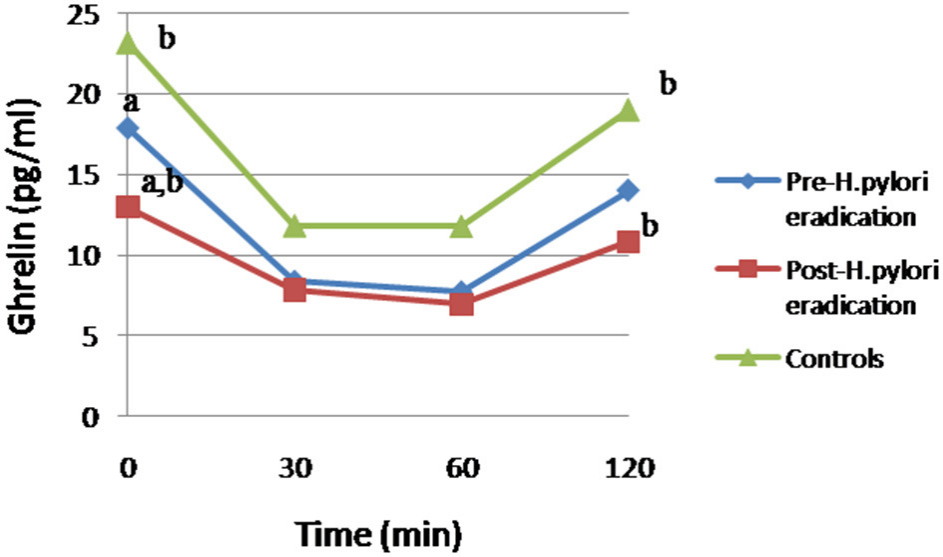
Changes in ghrelin levels after 75g-OGTT in patients and controls. Equal letter means differences between groups. (a) Differences between *H. pylori*-infected individuals before and after antibiotic treatment, *p* < 0.05. (b) Differences in patients undergoing antibiotic therapy compared to the control group, *p* < 0.05.

Focusing on ghrelin levels, after *H. pylori* eradication treatment, we have observed a statistically significant decrease of the fasting plasma ghrelin levels respect to patients pre-treatment (12.97 ± 9.51 vs. 17.82 ± 13.38, *p* = 0.017) and controls (12.97 ± 9.51 vs. 23.13 ± 19.27, *p* = 0.04; Figure 1). After the functional glucose test, the ghrelin AUC decreased significantly after the *H. pylori* eradication therapy (1,065.85 ± 550.87 vs. 1,273.73 ± 717.67, *p* = 0.03). However, no statistically significant differences were detected in the ghrelin AUC between patients (pre- and post-treatment) and controls (Figure 1). Interestingly, after 120 min of the ingestion of the glucose bolus, ghrelin levels remained significantly lower in post-treatment patients compared to controls (10.82 ± 8.67 vs. 18.99 ± 18.92, *p* = 0.039), but no statistically significant changes were detected between *H. pylori* positive patients (pre-eradication treatment) and controls.

To delve into these results, through a simple linear correlation analysis we have related BMI and ghrelin levels, indicating that the ghrelin AUC and fasting ghrelin levels were negatively associated with BMI (*r* = −0.289, *p* = 0.004 and *r* = −0.242, *p* = 0.017, respectively). Reinforcing these results, a univariate regression analysis, associated ghrelin AUC (*R*^2^ = 0.056, β = −0.238, and *p* = 0.019) and fasting ghrelin levels (*R*^2^ = 0.057, β = −0.239, *p* = 0.018) with BMI (as an independent variable).

### Fasting Ghrelin Levels and Gut Microbiota

In a previous study, we already reported alterations in richness, diversity (Chao 1 and Shannon indices, respectively) and specific bacteria related to *H. pylori* infection and eradication treatment in these subjects (17). Our study model in subjects without metabolic abnormalities or other diseases allows us to evaluate changes in specific bacteria and ghrelin levels, as well as the relationship between both variables, avoiding possible confounding factors associated with these variables.

**Figure 2:**
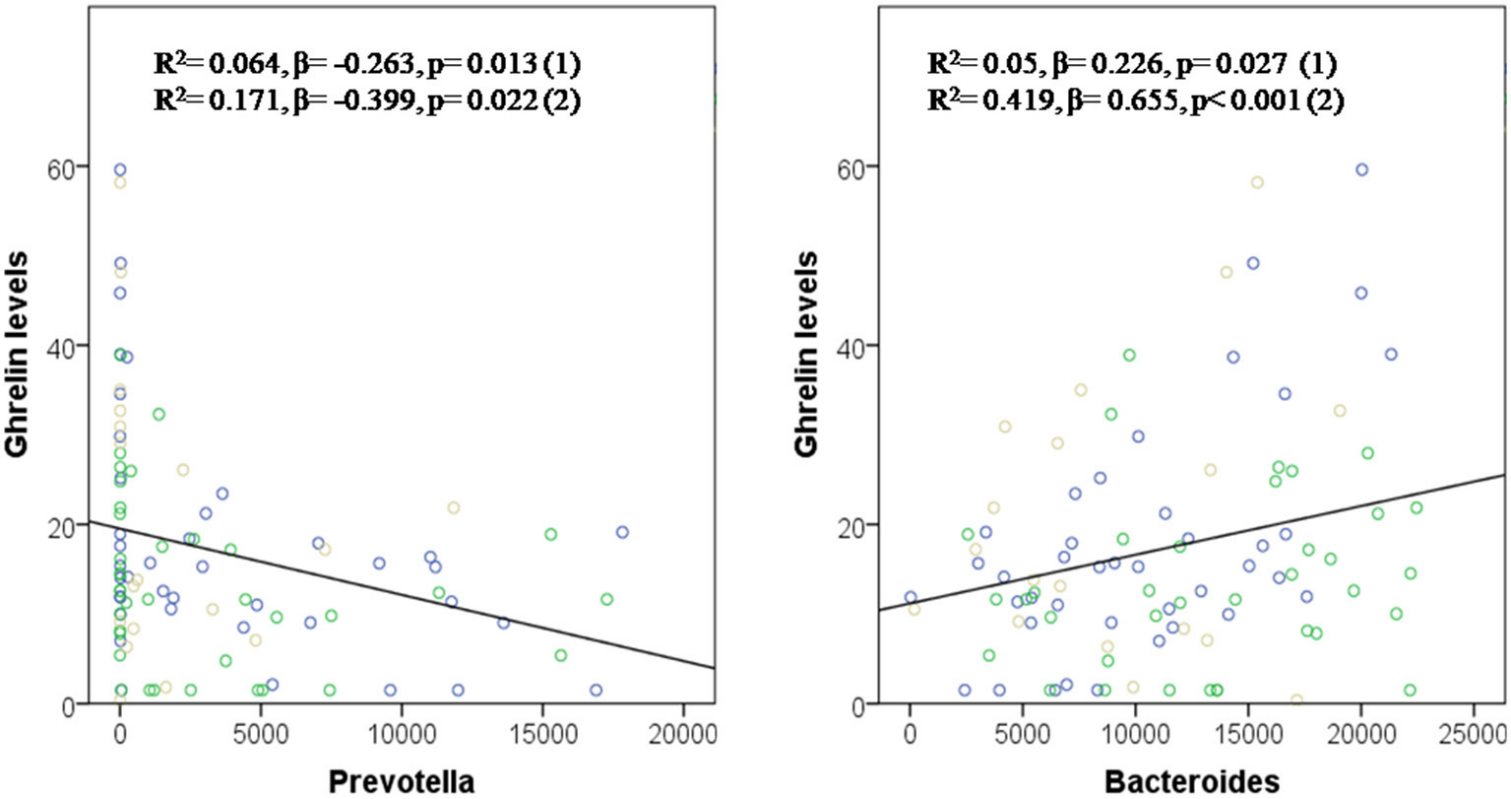
*Prevotella* and *Bacteroides* in the prediction of fasting ghrelin levels. Dependent variable: ghrelin levels. Model (1): Linear Univariate regression model. Model (2): Linear multivariate regression model adjusted by age, sex, and BMI. The brown circles belong to the control group; blue circles to patients before treatment; and green circles to patients after treatment.

A simple linear correlation analysis, which included the whole sample population (*H. pylori* positive, post-treatment and control), associated fasting ghrelin levels with specific intestinal bacteria (Table 1). In addition, these relationships between the different bacteria and ghrelin levels were explained by regression model. Thus, the abundance of *RS045* (*R*^2^ = 0.062, β = 0.249, and *p* = 0.015), *Bacteroides* (*R*^2^ = 0.05, β = 0.226, *p* = 0.027 and *R*^2^ = 0.419, β = 0.655, *p* ≤ 0.001 adjusted), *Bifidobacterium longum* (*R*^2^ = 0.070, β = 0.264, *p* = 0.010), and *Parabacteroides distasonis* (*R*^2^ = 0.043, β = 0.207, *p* = 0.044, and *R*^2^ = 0.192, β = 0.416, *p* = 0.014 adjusted) predicted positively fasting ghrelin levels, while the abundance of *Prevotellaceae* (*R*^2^ = 0.068, β = −0.260, *p* = 0.011 and *R*^2^ = 0.171, β = −0.399, *p* = 0.022 adjusted), its genus *Prevotella* (*R*^2^ = 0.064, β = −0.263, *p* = 0.013 and *R*^2^ = 0.171, β = −0.399, *p* = 0.022 adjusted), and its species *Prevotella stercorea* (*R*^2^ = 0.055, β = −0.235, *p* = 0.022) predicted negatively fasting ghrelin levels. Figure 2 graphically represents the association found between fasting ghrelin and genera.

**Table 1:** Linear correlation between bacteria and fasting ghrelin levels.

**Phyla**	**Families/Genera/Species**	**Ghrelin**	
		**Spearman's Rho**	***p*** **-value**
**Actinobacteria**	*Bifidobacterium longum*	0.219	0.033
**Firmicutes**	*Megasphaera*	−0.266	0.009
	*Roseburia faecis*	0.235	0.022
**Bacteroidetes**	*Bacteroides*	0.207	0.044
	*Bacteroides ovatus*	0.221	0.031
	*Bacteroides plebeius*	−0.216	0.035
	*Prevotellaceae*	−0.321	0.001
	*Prevotella*	−0.295	0.004
	*Prevotella stercorea*	−0.215	0.037
	*Rikenellaceae*	0.209	0.040
	*Parabacteroides distasoni*	0.240	0.019
**Saccharibacteria** (formed TM7)	*RS045*	0.274	0.007
**Proteobacteria**		0.228	0.026

*Spearman correlation test used to compare bacterial abundance and fasting ghrelin levels, including the 3 groups studied. Statistically significant data is shown (p ≤ 0.05)*.

### Post-meal Ghrelin Levels and Gut Microbiota

A significant univariate correlation between the abundance of specific bacteria and the post-meal ghrelin levels (referred to 120 min after the ingestion of the glucose bolus) were found. The analysis showed a significant positive correlation between *Streptococcus* (*r* = −0.217, *p* = 0.35), *Bacteroides ovatus* (*r* = 0.217, *p* = 0.36), *RS045* (*r* = 0.294, *p* = 0.004) and post-meal ghrelin levels, while *Bacteroides coprophilus* (*r* = −0.302, *p* = 0.003), *Megasphaera* (*r* = −0.234, *p* = 0.023) showed a significant negative correlation with post-meal ghrelin levels. In the univariate regression analysis, the genus *RS045* (*R*^2^ = 0.079, β = 0.282, and *p* = 0.006) predicted post-meal ghrelin levels.

### Bacterial Changes Associated With Changes in Post-treatment Ghrelin Levels

**Figure 3:**
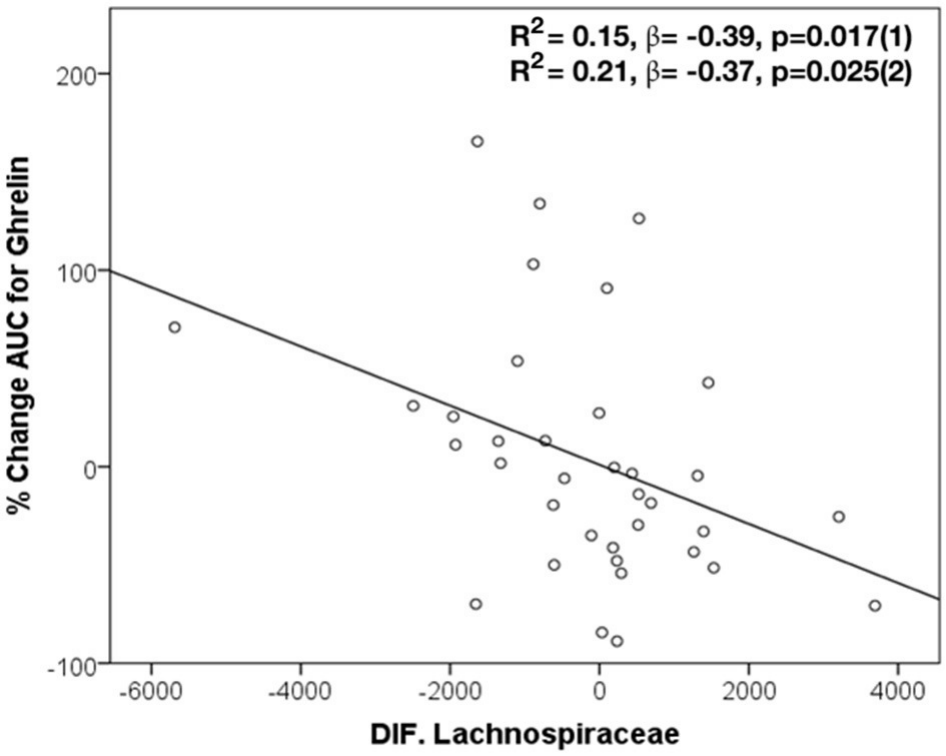
Changes in *Lachnospiraceae* in the prediction of modifications in AUC for ghrelin after *H. pylori* eradication treatment. Dependent variable: ghrelin AUC. Model (1): Linear Univariate regression model. Model (2): Linear multivariate regression model adjusted by age, sex, and BMI.

In this study, changes in the abundance of specific bacteria after *H. pylori* eradication treatment have been correlated with changes in the ghrelin AUC. Specifically, an inverse correlation between ghrelin AUC and changes in the abundance of *Lachnospiraceae* (*r* = −0.43, *p* = 0.008), its genus *Blautia* (*r* = −0.52, *p* = 0.001), and *P. stercorea* (*r* = −0.34, *p* = 0.038) after the triple therapy have been identified. In addition, through linear regression analysis, changes in *Lachnospiraceae* (*R*^2^ = 0.15, β = −0.39, *p* = 0.017 and *R*^2^ = 0.21, β = −0.37, *p* = 0.025 adjusted) predicted the proportion of changes in the ghrelin AUC, in patients after eradication treatment (Figure 3). On the other hand, changes in the abundance of *Megasphaera* were associated with changes in post-eradication fasting ghrelin levels (*r* = −0.374, *p* = 0.02).

## Discussion

Although it is known that *H. pylori* infection and its eradication therapy cause perturbations in the gut microbiome, in spite of the existence of several studies that point to metabolic changes in these patients, including alterations in BMI, how these disturbances are able to influence host metabolism is a little studied field. In this study, changes in the gut microbiota have been related to ghrelin levels in otherwise healthy *H. pylori*-infected subjects receiving the standard triple therapy.

Ghrelin levels are altered by the *H. pylori* eradication therapy. In fact, our study, in accordance to other studies (26, 31), have shown a decrease in plasma ghrelin levels after eradication therapy. In addition, other authors have shown a decrease in plasma desacyl-ghrelin levels (32) but an increase in plasma acyl-ghrelin levels (21, 32). The decrease in ghrelin levels could be attributed to the damages caused to ghrelin-producing endocrine cells by *H. pylori* (33, 34). However, different aspects point out that microbiota, a direct target of antimicrobials, could be partly involved in alterations observed in the ghrelin levels. First of all, the use of antimicrobials is known to alter the profile of the microbiota (35) and changes in the composition of the gut microbiota have been linked to ghrelin levels (14). Moreover, a mouse-study has demonstrated that *H. pylori* and gut microbiota-associated modulation of metabolic gut hormones was independent and preceded *H. pylori*-induced histopathological changes in the gut of infected mice (25). Finally, patients included in this study eradicated *H. pylori* post-therapy, and it has been reported that the successful *H. pylori* eradication treatment in subjects without severe gastric atrophy or intestinal metaplasia leads to improvements in gastric mucosal patterns in the short term (36). However, a plasma ghrelin reduction have also been observed in patients who failed *H. pylori* eradication therapy (26).

As mentioned in our previous study, *H. pylori* infection and its eradication with triple therapy decreased diversity and richness, as well as changed the abundance of specific bacteria, with persisting effects after 2 months of the *H. pylori* eradication treatment (17). In the current study, specific bacterial changes have been associated with both fasting and postprandial ghrelin levels. In line with our results, previous studies have positively associated ghrelin levels with *Bacteroides* (37, 38), *Bifidobacterium* (39), and *Parabacteroides* (40), and negatively with *Prevotellaceae* (41), *Blautia* (42), and *Streptococcus* (42). But data have also been reported in the other direction, with negative associations between ghrelin and *Bifidobacterium* (37, 43), *Bacteroides* (42) and positive with *Prevotella* (37, 44). However, most of these relationships have been found as the result of interventions with non-antibiotic therapies or in animals (14). To date, only one previous study has linked changes in ghrelin and gut bacteria (Bacteroidetes/Firmicutes) after *H. pylori* eradication therapy in humans (26). Therefore, our results could contribute to broadening the knowledge on the eradication therapy-gut microbiota-ghrelin relationship. Furthermore, many of the ghrelin-related taxa identified in our study are novel.

How gut microbiota is able to mediate its influence to the host is a matter of continuous study. Evidence suggests that microbial metabolites could be the mediators between the gut microbiota and host homeostasis. Among these metabolites, short-chain fatty acids (SCFAs), mainly acetate, propionate, butyrate, are known to exert multiorgan effects on the host energy metabolism (45). Therefore, microbial imbalance by *H. pylori* eradication treatment could affect the production of these bacterial metabolites, and consequently affect host metabolism, including ghrelin regulation. In fact, although more studies are necessary, changes in the microbiome and its metabolites have been related to alterations in ghrelin expression, secretion, activation, and signaling and appetite regulation through ghrelin (14).

Colonic SCFAs production can negatively affect serum ghrelin concentration (46) probably *via* SCFA receptors on enteroendocrine cells, as already shown for example for GLP-1 (47). Some studies have observed an increase in fecal butyrate and a decrease in plasma ghrelin (40, 46). Butyric acid is one of the most abundant and important SCFAs in the gut due to its multiple effects, like its participation in energy homeostasis by regulating appetite and energy intake (48). In addition, butyrate has been involved in ghrelin signaling (49). In our study, changes in the abundance of butyrate-producing bacteria such as *Lachnospiraceae* and *Megasphaera* have been inversely related with changes both in ghrelin AUC and fasting ghrelin levels after eradication therapy. Interestingly, changes in *Lachnospiraceae* after triple therapy predicted changes in ghrelin AUC, regardless of age, gender and BMI. *Lachnospiraceae* and *Megasphaera* are considered health promoting bacteria (50).

Lactate is another bacterial metabolite that can be produced by many intestinal bacteria, but its accumulation in the colon is often an indicator of microbiota perturbation or dysbiosis (51). Lactate is able to suppress ghrelin through the inhibition of the secretory function of ghrelin producing gastric cells, in addition to its possible involvement in modulation signaling through the ghrelin receptor (52). In our study, lactate-producing bacteria such as *Streptococcus* and *Blautia* were negatively associated with ghrelin. Interestingly, increase in *Blautia* after triple therapy was associated with decrease in ghrelin AUC. *Blautia* has been associated with metabolic disorders, including obesity (53), but it has also been inversely associated with visceral fat accumulation (54). The imbalance between lactate-producing and utilizing bacteria or a reduction in lactate-utilizing bacteria leads to dysbiosis. In fact, communities with low numbers of lactate-utilizing bacteria are inherently less stable and more prone to lactate-induced perturbations (51). *Lachnospiraceae* and *Megasphaera*, in addition to their ability to degrade complex polysaccharides to butyrate, are able to use lactate to generate propionate through the acrylate pathway (55), therefore, these bacteria could be categorized as lactate-utilizing bacteria. Other bacteria inversely related to ghrelin in our study, such as *Prevotella*, can also use lactate to produce succinate, a precursor to propionate (56), which could contribute to the reduction of colonic lactate levels, and therefore, increase ghrelin levels. However, propionate has been related to satiety by decreasing in ghrelin levels (57). On the other hand, propionate has been related to lipid metabolism (58), and in our previous study, propionate precursor bacteria such as members of *Lachnospiraceae* have been positively related to HDL cholesterol levels (19). Since, *Lachnospiraceae* was negatively associated with ghrelin in this study, and this hormone is involved in the regulation of lipid metabolism, promoting fat storage, in addition to exerting direct peripheral effects on lipid metabolism (59), we speculate that members of *Lachnospiraceae* could be related to lipid metabolism through propionate levels and variations in ghrelin levels.

In contrast, the lactate and acetate producer *Bifidobacterium longum*, belonging to the Actinobacteria phylum, predicted positively fasting ghrelin levels, in our study. *B. longum* has been previously linked to the ghrelinergic signaling, which is an important signaling pathway modulating central appetite regulation and metabolism (49). Specifically, *B. longum* may decrease the GHSR-1a internalization and has been correlated with its higher acetate content (49), but also with decrease body weight gain, fat depot size, glucose tolerance, and leptin levels in a preclinical mouse model of HFD-induced obesity (60). In the current study, we have observed a marked decrease in *Bifidobacterium*, including *B. Longum* at post-eradication therapy compared to controls and/or *H. pylori* positive (17) which could be contributing to the decline in nocturnal plasma ghrelin observed. Declining ghrelin levels contributes to this reduction in food intake and lean body mass (61). However, *H. pylori* eradication has been related to an increase in BMI (23). Although, we did not observe significant changes in BMI after eradication, possibly due to the short follow-up period, we observed a negative association between fasting ghrelin levels and BMI. These discrepancies could be related to the gut microbiota and its ghrelinergic effects previously shown (49). We speculate that the higher levels of ghrelin in controls and *H. pylori* positive together with the presence of *B. Longum* would have an attenuated response on its receptor and therefore on food intake and energy metabolism. Although Actinobacteria represent only a small percentage of the gut microbiota, this phylum is pivotal in the maintenance of gut homeostasis, and an unbalanced abundance has been evidenced in several pathological conditions (62).

Other acetate-producing bacteria, such as *Bacteroides, P. distasonis*, positively predicted fasting ghrelin levels regardless of age, gender, and BMI, and these bacteria have been associated with obesity (53). It has been suggested that acetate could be mediating changes in ghrelin signaling, which could indicate an interaction between the microbiota and ghrelin (13). In fact, increased acetate production by an altered gut microbiota leads to activation of the parasympathetic nervous system which in turn promotes increased glucose-stimulated insulin secretion (GSIS), increased ghrelin secretion, hyperphagia, obesity, and its related sequelae (13). It has also been suggested that the acetate reduces GHSR-1a internalization and acetate, propionate and lactate inhibit ghrelin-mediated receptor internalization (49).

Finally, we have observed that the abundance of *RS045*, belonging to the former TM7 phylum and currently known as *Saccharibacteria*, predicted fasting and post-meal ghrelin levels. TM7 has an ultra-small size and lives on the surface of its host bacterium, and lacks the ability to synthesize any of its own amino acids, vitamins, or cell wall precursors, thereby parasitizing other bacteria. TM7 has been strongly associated with all adiposity markers (63).

In the present study, there are several limitations that must be taken into consideration. *H. pylori* could intervene in ghrelin secretion and therefore be a confusing factor. However, the strength of the current study is the fact that it was performed in otherwise healthy patients with no other confounding variables. Although, the inclusion of a group of subjects without *H. pylori* infection exposed to eradication treatment could have provided more detailed information on the role of antibiotic treatment in the association found, it was not possible for ethical reasons. In this manner, it would be also of interest to study the alterations produced by the different drugs used in the *H. pylori* eradication therapy separately, but since it is a procedure not validated in clinical practice, this approach only could be carried out in the form of a particular clinical trial for ethical reasons. On the other hand, the sample size could be increased, although previous calculations of the sample size were performed to ensure a realistic approach. Lastly, the 16s ribosomal RNA gene sequencing used has limitations in identifying genetically specific species and strains.

In summary, eradication treatment for *H. pylori* could decrease ghrelin levels, and this alteration could be mediated through the changes produced in the gut microbiota composition by the antibiotic therapy. These results could indicate that the imbalance between lactate producers and its utilizers could trigger changes related to ghrelin levels under alteration of *H. pylori* eradication therapy. In addition, acetate, and butyrate producing bacteria identified in the study could also play an important role in ghrelin regulation. We suggest that ghrelin secretion and signaling may be under regulation by intestinal commensal microbes and affected by antibiotics. More studies are required to clarify the effect of the antibiotic on ghrelin.

## Data Availability Statement

The datasets presented in this study can be found in online repositories. The names of the repository/repositories and accession number(s) can be found below: https://www.ncbi.nlm.nih.gov/ (PRJNA517270).

## Ethics Statement

The studies involving human participants were reviewed and approved by Medical Ethics Committee at Virgen de la Victoria University Hospital. The patients/participants provided their written informed consent to participate in this study.

## Author Contributions

GMM-N performed the metagenomic analysis, statistical analysis and interpretation of data, drafting, and reviewing of the manuscript. IC-P performed the recruitment follow-up of the patients, and revision of the manuscript. MC-P performed laboratory analysis and was involved in the revision of the manuscript. FJT contributed to the study concept and design, interpretation of data, reviewed, and critically revised the article for important intellectual content. IM-I contributed to the study concept, interpretation of data, bioinformatic analysis, and critical revision of the manuscript. All authors contributed to the article and approved the submitted version.

## Conflict of Interest

The authors declare that the research was conducted in the absence of any commercial or financial relationships that could be construed as a potential conflict of interest.

## Publisher's Note

All claims expressed in this article are solely those of the authors and do not necessarily represent those of their affiliated organizations, or those of the publisher, the editors and the reviewers. Any product that may be evaluated in this article, or claim that may be made by its manufacturer, is not guaranteed or endorsed by the publisher.
